# The Role of Zinc in Developed Countries in Pediatric Patients: A 360-Degree View

**DOI:** 10.3390/biom14060718

**Published:** 2024-06-17

**Authors:** Flavia Padoan, Elena Piccoli, Angelo Pietrobelli, Luis A. Moreno, Giorgio Piacentini, Luca Pecoraro

**Affiliations:** 1 Pediatric Unit, Department of Surgical Sciences, Dentistry, Gynecology and Pediatrics University of Verona, 37126 Verona, Italy; 2 Growth, Exercise, Nutrition and Development (GENUD), Research Group, Instituto Agroalimentario de Aragón (IA2), Instituto de Investigación Sanitaria Aragón (IIS Aragón), Universidad de Zaragoza, 50001 Zaragoza, Spain

**Keywords:** zinc, prevention, supplementation, infections, anti-inflammatory, antioxidant

## Abstract

Zinc is an important trace element for growth and health at pediatric ages. Zinc is fundamental in inflammatory pathways, oxidative balance, and immune function. Zinc exhibits anti-inflammatory properties by modulating Nuclear Factor-kappa (NF-κB) activity and reducing histamine release from basophils, leukocytes, and mast cells. Furthermore, its antioxidant activity protects against oxidative damage and chronic diseases. Finally, zinc improves the ability to trigger effective immune responses against pathogens by contributing to the maturation of lymphocytes, the production of cytokines, and the regulation of apoptosis. Given these properties, zinc can be considered an adjunctive therapy in treating and preventing respiratory, nephrological, and gastrointestinal diseases, both acute and chronic. This review aims to deepen the role and metabolism of zinc, focusing on the role of supplementation in developed countries in pediatric diseases.

## 1. Introduction

Zinc is an important trace element for human health because it is involved in numerous biological processes throughout the body, including cell growth and differentiation, gene expression, and protein synthesis [1]. The main sources of zinc intake are dairy products, seafood, meat, and poultry [2] (Table 1). Absorption occurs in the intestine and eventual excretion in stool [3]. Zinc bioavailability depends on the composition of the diet. Non-digestible plant ligands such as phytates, certain dietary fibers, and lignin can chelate zinc and inhibit its absorption. Other factors that influence zinc absorption are calcium and iron [4]. The dietary reference intake (DRI) for zinc in infants is 2.9 mg/day, rising to 4.3 mg/day in toddlers. In adolescence, the DRI is 11 mg/day [5,6]. The human body contains about 2–4 g of zinc [4]. Zinc is present in all tissues, particularly in skeletal muscle and bone, which contain about 83% of the total zinc in the body. On the other hand, a smaller portion is present at the plasma level, bound mainly to albumin. This accounts for about 0.1% of total body zinc (14 μmol/L) [1,7]. About 90% of zinc is involved in cell maintenance functions as a structural element stabilizing DNA-binding domains of transcription factors and metalloenzymes [8]. In contrast, a smaller amount constitutes labile zinc pools, which regulate organ-specific zinc-dependent processes, including signal transduction, apoptosis, secretion, fertilization, and neurotransmission [9,10]. These labile pools are rapidly depleted upon zinc deficiency (ZnD), while the amounts of zinc involved in structural functions generally remain unaffected [11]. The International Zinc Nutrition Consultative Group (IZiNCG) has suggested using a serum concentration value < 60–70 ug/dL as the cut-off for defining ZnD [12]. However, since serum zinc concentration is only a small percentage of the total amount of zinc at the body level, it may not reflect body-level zinc reserves. Therefore, assessment of serum zinc levels is not recommended for routine screening [13]. Therefore, other biomarkers have been evaluated that could be valuable elements to assess zinc status in the future, such as, for example, urinary zinc excretion, zinc concentration in hair, or zinc-binding proteins, such as metallothionein (MT). The results in the literature regarding these biomarkers are still controversial [14,15,16,17,18]. In addition, a presumptive diagnosis of ZnD can be made based on the presence of symptoms of zinc deficiency, signs of malnutrition, or conditions commonly associated with ZnD [6,13,19,20,21,22].

ZnD particularly impacts developing countries and contributes to various health issues. In low- and middle-income countries, varying prevalence rates of ZnD have been reported in infants, young children, and preschool-age children, ranging from 5.1% in Sri Lanka to 82.6% in Cameroon [23]. According to the World Health Organization (WHO), ZnD is the fifth most important underlying factor for deaths in developing countries [24]. Populations in low- and middle-income countries are at an increased risk of inadequate zinc intake, which can be partially attributed to limited access to foods rich in zinc, such as animal products, in combination with a mainly plant-based diet, which contains phytates that inhibit intestinal zinc absorption [25]. In developing countries, where malnutrition is often prevalent and access to adequate healthcare is limited, zinc plays a crucial role in public health and development [26]. The implications of ZnD in these regions are multifaceted and far-reaching, impacting individual health and national development efforts. In these countries, children are particularly vulnerable to ZnD, leading to stunted growth, impaired cognitive development, and increased susceptibility to infectious diseases [27,28]. These consequences perpetuate a cycle of poverty and underdevelopment as malnourished children struggle to reach their full potential, both physically and intellectually. Moreover, ZnD has profound implications for maternal health and reproductive outcomes [29,30]. Deficient zinc levels during pregnancy can increase the risk of complications, such as preterm birth, low birth weight, and congenital abnormalities, further exacerbating the cycle of underdevelopment [31]. In addition, zinc plays a key role in supporting immune function [21], so its deficiency impairs the body’s ability to fight infection, contributing to high morbidity and mortality rates [32]. Conversely, for example, zinc supplementation combined with oral rehydration therapy has been shown to play a role in reducing the duration and severity of acute diarrhea episodes, an important cause of morbidity and mortality, especially among children in developing countries [33].

**Table 1:** Zinc content in most common foods. Modified from [34].

Food	Serving	Milligrams (mg) of Zinc	DRI%
Seafood			
Oysters	85 g	32	291
Shrimps	85 g	1.4	13
Sardines	85 g	1.1	10
Salmon	85 g	0.5	5
Meat			
Beef, bottom sirloin, roasted	85 g	3.8	35
Pork, chops	85 g	1.9	17
Turkey breast	85 g	1.5	14
Peanuts	28 g	0.8	7
Bread, cereals, legumes, and seeds			
Whole wheat bread	1 slice	0.6	5
White bread	1 slice	0.2	2
Cereals, unenriched oats cooked with water	1 cup	2.3	21
Brown rice, cooked	½ cup	0.7	6
White rice, cooked	½ cup	0.3	3
Lentils, boiled	½ cup	1.3	12
Kidney beans	½ cup	0.6	5
Pumpkin seeds	28 g	2.2	20
Milk and dairy products			
Milk	1 cup	1.0	9
Cheese, cheddar	42.5 g	1.5	14
Greek yogurt	170 g	1.0	9
Fruits and vegetables			
Broccoli	½ cup	0.4	4
Cherry tomatoes	½ cup	0.1	1
Blueberries	½ cup	0.1	1
Other			
Egg	1 unit	0.6	5

Addressing ZnD in developing countries requires a comprehensive approach that includes nutritional interventions, such as increased accessibility to zinc-rich foods and fortified staple foods, improvements in health infrastructure, and socioeconomic empowerment strategies [35,36,37]. Increasing the DRI from two to five for six months is recommended in case of zinc deficiency, depending on the severity. For acute diarrhea in malnourished children aged 6 to 36 months, 5 to 20 mg per day of elemental zinc has been used. The maximum tolerable intake level varies based on age and is between 4 mg in newborns and 40 mg in adults. The dose should not exceed the maximum tolerable intake level for prolonged periods [13]. Although reported in a few studies, ZnD problems also affect healthy children in high-income countries, in which prevalence rates of 0 to 60% have been identified [38,39,40,41,42].

### 1.1. Role of Zinc in Human Biological Pathways

Zinc plays a vital role in numerous cellular activities, including cell signaling, differentiation and growth, maintaining homeostasis and immune responses, managing oxidative stress, and protecting against antioxidants. It is essential for processes like apoptosis and aging [43]. This mineral is a key component in over 300 enzymes, such as hydrolases, transferases, oxidoreductases, ligases, isomerases, and lyases. Zinc is crucial for the stability and integrity of cell membranes and ion channels. It also acts as an intracellular regulator, structurally supporting proteins during molecular interactions, and serves as a structural component in nucleic acids and other proteins involved in gene regulation [44].

Two important families of proteins that use zinc as a cofactor to carry out their biochemical roles are metallothioneins (MTs) and zinc-finger proteins (ZFPs).

MTs are cysteine-rich, low-molecular-weight proteins crucial for binding heavy metals, maintaining metal homeostasis, and detoxifying cells. They protect against metal toxicity and oxidative stress. In children, MT dysregulation can lead to diseases such as neurological disorders, developmental delays, and metabolic syndromes. MTs play a significant role in neurodevelopment by binding essential metals like zinc and copper. Imbalances in these metals are linked to neurodevelopmental disorders like autism spectrum disorder (ASD) [45], where altered MT levels can cause oxidative stress and neuroinflammation. MTs also play roles in genetic disorders involving copper metabolism, such as Wilson’s disease and Menkes disease [46,47,48]. In Wilson’s disease, mutations cause copper accumulation, leading to liver and neurological issues, while MT dysregulation exacerbates the condition. Menkes disease, caused by different mutations, results in copper deficiency, affecting brain development. Proper MT functioning is crucial for managing these conditions. MTs also protect against heavy metal toxicity, such as lead and cadmium exposure, which can cause cognitive impairments and developmental delays in children [49]. By binding these metals, MTs reduce their toxic impact. Additionally, MTs regulate the immune response and inflammation, with dysregulation potentially leading to autoimmune conditions. Maintaining proper MT levels in children is essential for balanced immune function and overall health [50]. 

ZFPs are characterized by zinc-finger domains stabilizing their structure. They are key regulators of gene expression and cellular functions, including DNA recognition, RNA binding, and protein–protein interactions. In children, mutations or the dysregulation of ZFPs can lead to significant developmental and health issues. For instance, ZNF143 regulates genes critical for cell proliferation and differentiation, with mutations linked to developmental disorders and cancers such as leukemia and neuroblastoma [51,52]. ZNF41 mutations cause X-linked intellectual disability (XLID), leading to developmental delays and intellectual disabilities [53]. The Wilms’ tumor protein (WT1) is a ZFP essential for kidney and gonadal development, with mutations causing Wilms’ tumor and syndromes like Denys–Drash and Frasier, characterized by nephropathy and gonadal dysgenesis [54]. Understanding ZFPs’ roles in gene expression and cellular function helps elucidate the molecular mechanisms underlying various developmental disorders and cancers, highlighting the importance of research for developing targeted therapies.

#### 1.1.1. Anti-Inflammatory Role of Zinc

Zinc is important for its anti-inflammatory role. Zinc modulates inflammatory cascades both upstream, through the modulation of Nuclear Factor-kappa (NF-κB) [55,56], and downstream, by acting on the effector cells of the inflammatory process. NF-κB is a transcription factor involved in regulating various immune and inflammatory responses. In unstimulated cells, NF-κB proteins are sequestered in the cytosol through interactions with a class of inhibitory proteins called IκB. Many stimuli induce NF-κB activity, including tumor necrosis factor (TNF), interleukin-1 (IL-1), protein kinase C (PKC) activators, viruses, bacterial lipopolysaccharides (LPSs), ionizing radiation, and oxidants [57]. These signals cause phosphorylation and subsequent degradation of IκB proteins through the ubiquitination-proteasome pathway, and as a result, free NF-κB can enter the nucleus and induce gene expression [58]. Zinc plays a crucial role in modulating NF-κB activity through several mechanisms. Zinc can directly inhibit NF-κB activation by interfering with the binding of NF-κB to DNA, thus inhibiting its transcriptional activity [59]. In addition, zinc induces the expression of MTs, cysteine-rich proteins capable of binding to zinc ions. MTs sequester free zinc ions, preventing their involvement in redox reactions that could activate NF-κB signaling. By sequestering zinc, MTs indirectly inhibit NF-κB activation [60]. Zinc can also modulate the activity of kinases involved in NF-κB activation. For example, zinc inhibits the activity of IκB kinase (IKKβ), which phosphorylates the NF-κB inhibitor (IκB), leading to its degradation and subsequent release of NF-κB for nuclear translocation [56]. Zinc also has antioxidant properties, as it scavenges free radicals and inhibits the production of reactive oxygen species (ROS) [56]. Since oxidative stress can activate NF-κB signaling, the antioxidant activity of zinc indirectly inhibits NF-κB activation. In addition, ZFPs aid in the transcription and stability of these proteins, some of which are regulators of NF-κB activity [60,61]. The downstream anti-inflammatory action of zinc occurs by acting on the effector cells of factors that contain zinc ions in their structure. Zinc availability influences the function of the inflammatory process in areas such as basophils, leukocytes, and mast cells. When activated, these cells can release histamine, a biogenic amine involved in immune responses, particularly in allergic and inflammatory reactions. However, several studies have shown that physiological concentrations of zinc inhibit this release because this ion acts as a competitive Ca^2+^ antagonist in antigen- and anti-IgE-induced histamine secretion [62]. In this regard, it has indeed been observed that ZnD increases allergic inflammation and is a risk factor for the development of asthma [63,64]. Therefore, several studies have analyzed this mechanism and observed that dietary zinc supplementation can alleviate allergic symptomology [65] and has a promising role in reducing airway hyper-responsiveness when administered before allergen exposure [66]. Additional evidence suggests that zinc modulates several aspects of inflammation, including serotonin release from platelets [67], macrophage and neutrophil phagocytosis [68], lymphocyte proliferation [69], and immune hemolysis [70]. 

#### 1.1.2. Antioxidant Role of Zinc

Another important role of zinc in the human body is as an antioxidant, even if it does not fit neatly into the traditional definitions of antioxidants (any substance that hinders a free radical reaction). Its antioxidative mechanism can be categorized into acute and chronic effects [61]. Chronic exposure to zinc induces MT in different organs, such as the liver, kidney, and intestine [71]. These low-molecular-weight proteins act as antioxidants binding bivalent or univalent metals. MT can be divided into different classes: MT-1 and MT-2 are ubiquitous throughout the body, while the expression of MT-3 and MT-4 is limited to specific cell types. MT-3 is predominantly found in the brain, and MT-4 is located primarily in stratified epithelial tissues [72]. Their main function is maintaining cellular zinc homeostasis and chelating heavy metals to reduce cytotoxicity and lower their intracellular concentrations [73]. Due to their reactive oxygen species scavenging properties, they help protect against several types of environmental stress. Several studies exhibit MT antioxidant effects in various conditions, such as radiation exposure [74], chemotherapy toxicity [75,76], and oxidative stress-induced mutagenesis [77]. In particular, under conditions of high oxidative stress, zinc is released from MTs due to sulfide/disulfide exchange. The sulfur clusters that bind zinc in MT create an oxidoreductive environment for zinc at a redox potential so low that MT can be readily oxidized by mild cellular oxidants, such as glutathione disulfide, with the release of zinc. This MT oxidation and zinc release process is critical in the cellular response to oxidative stress [78]. Conversely, chronic ZnD increases sensitivity to oxidative stress. On the other hand, the acute antioxidant effects of zinc involve short-term increases in its levels. Two main mechanisms are described: sulfhydryl stabilization and reduction in the formation of hydroxyl radicals from hydrogen peroxide (H_2_O_2_) and superoxide radical (O_2_^−^) by antagonizing redox-active transition metals like iron and copper [61]. Zinc stabilizes sulfhydryl groups by directly binding or inducing conformational changes in nearby proteins, reducing their reactivity [79]. It also interferes with the catalytic properties of iron and copper, which can initiate destructive lipid peroxidation processes [80]. Metal-catalyzed hydroxyl radical (^•^OH) formation can result in hydrogen abstraction from an unsaturated fatty acid, leading to lipid radical formation. Another possible site of attack can include proteins [81]. Zinc can compete with copper or iron for certain types of binding sites, and the net result is the displacement of the metal into the cytosolic compartment, where it can undergo hydrolytic polymerization and precipitation as an unreactive polynuclear structure [82,83] or possibly redistribute it to some other less critical site, thus shifting the site of formation of ^•^OH. Furthermore, zinc is an important constituent of superoxide dismutase (SOD) [84]. This enzyme is part of the body’s antioxidant defense system, along with catalase and glutathione peroxidase. SOD specifically targets O_2_^−^, converting it into H_2_O_2_ and O_2_. This process helps protect cells from oxidative damage caused by ROS. There are several types of SOD found in living organisms, classified based on the metal cofactor they contain. These include copper–zinc SOD (Cu/Zn-SOD), manganese SOD (Mn-SOD), and iron SOD (Fe-SOD) [7]. In Cu/Zn-SOD, zinc acts as a cofactor, binding to the enzyme and assisting in its catalytic activity. Zinc helps stabilize the structure of SOD, facilitating the conversion of superoxide radicals into less harmful substances. Without zinc, SOD cannot function optimally, leading to increased oxidative stress and potential damage to cells [85]. 

#### 1.1.3. Anti-Apoptotic Role of Zinc

Excessive oxidative stress can initiate the apoptotic process of programmed cell death, which involves several signaling pathways [86]. The mitochondrion is an important player in the induction, regulation, and execution of apoptosis under the control of anti-apoptotic (e.g., Bcl-2) and pro-apoptotic (e.g., BAX) regulatory genes [87]. Zinc can regulate the apoptotic process upstream by modulating the expression and activity of Bcl-2 family proteins [10]. Furthermore, it can inhibit the main signaling pathways involved in the apoptotic process by directly or indirectly influencing the activity of initiator caspases, such as caspase-9, and effector caspases, such as caspase-3 [10]. Several studies have shown that zinc localizes with the zymogenic form of caspase-3 in the apical cytoplasm of human airway epithelial cells and can directly prevent its activation. Consequently, a reduction in zinc levels can be associated with an increase in the active form of caspase-3, resulting in greater apoptotic activity [88]. 

#### 1.1.4. Role of Zinc in Immune Response

Finally, zinc plays an important role in modulating the immune response. Zinc plays a role in lymphocyte maturation, cytokine and ROS production, and apoptosis regulation. Therefore, ZnD impairs the function of various immune cells, including neutrophils, macrophages, natural killer (NK) cells, and T lymphocytes [89], leading to increased susceptibility to infections. ZnD causes an imbalance between Th1 and Th2 functions, reducing the activity of Th1 lymphocytes. Th1 lymphocytes produce interleukin-2 (IL-2) and interferon-gamma (IFN-γ), which are reduced during ZnD, while IL-4, IL-6, and IL-10 are not affected when produced by Th2 lymphocytes [90]. Furthermore, ZnD reduces the lytic activity of NK cells and influences the development and function of cytolytic T cells, including cytotoxic T lymphocytes (CTLs) and natural killer T cells (NKTs). These cells are an integral part of innate and cell-mediated immunity, which is fundamental for identifying and eradicating infected cells and initiating an effective immune response against pathogens. The alteration of zinc status significantly influences the immune response, increasing susceptibility to inflammatory and infectious diseases, including acquired immunodeficiency syndrome, measles, malaria, tuberculosis, and pneumonia [89]. By analyzing the anti-inflammatory, antioxidant, and immune role played by zinc at pediatric ages, we are now proposing an overview of the organs and systems that can be affected by zinc deficiency and in which pathologies possible zinc supplementation could play a role in prevention and treatment.

### 1.2. Role of Zinc in Human Organs and Systems

#### 1.2.1. Respiratory Diseases

Zinc has been studied in the context of bacterial infections, particularly in children with community-acquired pneumonia (CAP). CAP is a prevalent respiratory tract infection with high mortality rates in children under 5 years of age [91,92]. Saied et al. demonstrated that zinc supplementation, as an adjunctive therapy, reduced the length of hospital stay [93] and the duration of lung effusion in children with pneumonia under 5 years of age [94]. These advantages are due to its antibacterial and anti-inflammatory properties, as well as its role in regulating tissue growth and reducing the stimulus to the production of toxins by microorganisms [95,96,97]. In addition, ZnD causes involution of the thymus and the depression of lymphocyte proliferation and IL-2 production [98,99,100]. These mechanisms are less clear, and the effect of zinc supplementation on children with pneumonia is controversial in the literature. Yuan et al. demonstrated that zinc supplementation can increase serum zinc levels to normal values in infants with zinc deficiency, but the normalization of zinc levels with zinc supplementation did not improve the clinical outcomes of infants with pneumonia [101]. Furthermore, it improved immune status, including enhanced cell-mediated immune response, serum thymulin activity, and IL-2 production [102,103,104,105]. The activity of thymulin, necessary for the development of IL-2-producing T lymphocytes, depends on the presence of zinc in the peptide structure of thymulin [106]. The relationship between serum zinc concentration and lung function in the pulmonary field has also been studied in children with cystic fibrosis. ZnD may be common in pediatric patients due to impaired protein intake and fat malabsorption [107]. It has been documented in infants identified by newborn screening before initiating pancreatic enzyme therapy [108]. The main consequences of chronic ZnD are stunted growth, delayed sexual maturation, immune disorders, poor appetite, and diarrhea, each of which is frequently present in patients with cystic fibrosis [109,110]. The literature on the relationship between serum zinc concentrations and cystic fibrosis-related disorders such as malabsorption, poor growth, and impaired lung function is somewhat controversial [108,111,112,113,114,115,116,117]. A few studies in pediatric patients have found no correlation between zinc levels and nutritional or pulmonary status [112,114]. Other studies, however, have found an alteration in zinc status in patients with severe lung disease and moderate-to-severe growth retardation [115,116,117]. Low plasma zinc levels in cystic fibrosis were also accompanied by low IL-2 plasma levels, impaired NK cell activity, and low thymulin activity [113] (Table 2). Abdulhamid et al. suggested that supplementation with 30–45 mg/day of zinc may positively affect growth and lung function. In comparison, a dose lower than 30 mg/day may not have been sufficient to influence these outcomes due to possible non-optimal absorption of zinc from the gastrointestinal tract [118]. Assessing zinc status in patients with cystic fibrosis is essential because, as underlined by Damphousee et al. almost 25% of adults affected by cystic fibrosis, despite having a satisfactory nutritional status, have low levels of zinc in plasma correlated with worse clinical outcomes [119]. Similarly, Bauer et al. identified low serum zinc levels in one-third of children with cystic fibrosis in the first 3 years of life [120]. Another interesting topic concerns zinc supplementation in extremely low-birth-weight (ELBW) infants (<1000 g) with chronic lung disease (CLD). CLD, also known as bronchopulmonary dysplasia, is a common complication affecting up to 40% of ELBW newborns [121]. Newborns with CLD have high rates of postnatal growth failure [122,123] and neurodevelopmental impairments [124]. Poor growth in this population may be partially related to ZnD for two reasons. The first is linked to the high demand for zinc, which is caused by the rapid growth rate of preterm newborns [125,126,127]. The second is because in the fetal period, the greatest accumulation of zinc occurs in the third trimester [128,129,130]. Premature infants also have immature intestinal tracts and kidneys, increasing zinc losses [131,132]. An additional risk factor for zinc deficiency is related to the dramatic decrease in zinc content in breast milk during the first weeks of breastfeeding, regardless of maternal zinc status [133,134,135]. Shaikhkhalil et al. observed improved growth in ELBW infants with CKD receiving enteral zinc supplementation with zinc acetate (100% to 150% increase compared to baseline zinc intake) [136]. Zinc may compete with iron and copper for absorption sites, but Walravens et al. [137]. demonstrated that zinc supplementation did not influence the status of copper or iron when administered in doses up to 4.5 mg/day in infants. The role of zinc in modulating and enhancing the immune response attracted much interest during the recent SARS-CoV-2 pandemic. SARS-CoV-2 mainly affects the respiratory system, causing pneumonia and acute respiratory distress syndrome (ARDS) [138], whose management may require mechanical ventilation [139] with higher mortality rates [140]. Furthermore, advanced age and pre-existing chronic metabolic conditions such as diabetes, cardiovascular disease [141], and obesity [142] are also known risk factors for increased susceptibility and mortality from SARS-CoV-2. Older individuals are particularly vulnerable due to impaired immune function [143]. SARS-CoV-2 infection is less severe in children than adults and usually only requires supportive therapy. At pediatric ages, severe disease includes acute respiratory failure in children with comorbidities and a delayed hyperinflammatory response called multisystem inflammatory syndrome (MISC) in children with cardiac dysfunction [144]. SARS-CoV-2 infection significantly reduces the frequency of ciliary beats, compromising mucociliary clearance and increasing susceptibility to bacterial co-infections. Adequate zinc levels can reduce cytoplasmic manganese, which exerts a toxic effect on *S. pneumoniae* [145] by interrupting cell growth [146]. Furthermore, zinc increases the susceptibility of bacteria to killing by neutrophils [147]. Several studies have used zinc oxide nanoparticles to study their antibacterial effect [148] and have demonstrated an inhibition of the growth and biofilm formation of *S. Pneumoniae* [149] and other bacterial agents involved in the aetiology of pneumonia, including *K. Pneumoniae* [150], *S. Aureus* [151], and *P. Aeruginosa* [152]. Zinc supplementation can increase ciliary length in the bronchial epithelium of zinc-deficient rats and increase ciliary beating frequency in vitro [153,154]; thus, it may theoretically help alleviate SARS-CoV-2-induced mucociliary clearance dysfunction. Furthermore, zinc can regulate the expression of tight junction proteins such as zonula occludens-1 (ZO-1) and claudin-1 [155], improving barrier functions in the respiratory tract. Another mechanism through which zinc can be useful in the management of the virus is the inhibition of the RNA polymerase activity of SARS-CoV-2, decreasing its replication [156], but also the modulation of angiotensin-converting enzyme 2 (ACE2) receptors, which are essential for the entry of SARS-CoV-2 into target cells [157,158] (Table 2). Finally, zinc stimulates lymphocytes to produce interferon-alpha (IFN-α) [159] with the activation of the signaling pathway downstream of JAK1/STAT1 and the production of antiviral enzymes such as latent ribonuclease (RNase L) and RNA-activated protein kinase (PKR) [160]. As discussed previously, zinc deficiency causes increased expression and production of NF-κB in the lungs, resulting in the upregulation of target genes IL-1β, TNFα, and ICAM-1 (intercellular adhesion molecule 1) [161], stimulating inflammation locally (pneumonia) and at a systemic level (cytokine storm) [162]. These mechanisms highlight a potential role for zinc in modulating the body’s response to the virus. However, it is unclear whether supplementation in non-zinc-deficient individuals meeting the recommended daily dose can improve the immune response to pathogens [163]. Children with low serum zinc levels were found to have a higher number of hospitalizations, but no association was found between the severity of SARS-CoV-2 disease and serum zinc levels in children [164]. However, according to Doğan et al. since there is no defined treatment protocol for SARS-CoV-2 infection in children yet, zinc supplementation can be used as a supportive treatment in SARS-CoV-2 infection [165].

#### 1.2.2. Gastroenterological and Liver Diseases

Zinc plays an important role in growth and improves food intake in young children [166,167]. It can increase ghrelin secretion from the stomach, plasma ghrelin concentrations, and intestinal insulin-like growth factor (IGF-1) [167]. Consequently, due to the effect of these hormones on appetite [168], zinc contributes to increasing hunger and food intake and consequently causes weight gain and growth [169,170]. However, several studies have shown that zinc supplementation is effective in increasing growth regardless of food intake [167,171,172,173,174]. Therefore, zinc likely exerts its effect through a more complex mechanism that needs to be fully explained. One hypothesis is that zinc may improve protein synthesis [167] and improve the nutritional and muscular status of people suffering from malnutrition (Table 2). Zinc supplementation may also be suggested during gastrointestinal infections in pediatric patients. Particularly in the case of diarrhea in children aged 6 months or older, additional treatment with zinc has been shown to reduce the duration of the disease and decrease the likelihood of progression to persistent diarrhea, especially in children with signs of malnutrition [175]. Therefore, the WHO and UNICEF recommend supplementation with 20 mg of zinc to be administered daily for 10–14 days during diarrheal episodes, together with saline solutions for oral rehydration [176]. Multiple mechanisms determine the beneficial effect of zinc on gastrointestinal infections in pediatric patients. Several studies conducted on different intestinal diseases, such as salmonellosis and shigellosis, have shown that zinc plays an important role in the regeneration of the intestinal mucosa and in improving the absorptive and barrier functions of the mucosa. Zinc can contribute to protein synthesis and the inhibition of apoptosis [10], helping to repair damage to epithelial cells, stimulate proliferation, and improve resistance to apoptosis. These changes ultimately manifest as increased villus height, an increased ratio of villus height to crypt depth, and decreased intestinal permeability [177,178,179]. Furthermore, combined therapy with zinc supplementation in addition to conventional treatment, such as antidiarrheal drugs and probiotics, studied in bacterial and viral enteritis, increased flora diversity and abundance compared to traditional treatment alone. A study on rotavirus enteritis reported that conventional therapy is adequate to correct flora disorders and reduce the abundance of various harmful bacteria. However, combination therapy not only effectively inhibits the proliferation of conditionally pathogenic bacteria to maintain the stability of the gut microbiota ecosystem but also significantly increases the diversity and abundance of some beneficial groups of bacteria. Further, the presence of these groups was negatively correlated with relevant inflammatory factors due to their ability to exert anti-inflammatory effects on the intestinal mucosa, modulating leukocyte recruitment and reducing proinflammatory cytokines and mediators such as INF-α, nitric oxide (NO), IL-2, IL-6, and TNF [180,181]. Zinc supplementation can have a role in individuals with Wilson’s disease, especially in presymptomatic and neurologic patients [182]. The rationale behind this therapy is that zinc inhibits the intestinal absorption of copper and stimulates the synthesis of intestinal MT, which has a high affinity for copper and prevents the serous transfer of copper into the blood [183]. Being nontoxic and nonteratogenic, zinc can be administered at any age; however, further studies are needed to expand the current understanding of the utility and limitations of zinc therapy for Wilson’s disease in the pediatric population.

#### 1.2.3. Otological Diseases

Zinc supplementation can have a role in preventing otitis media and its complications. Specifically, it can reduce the incidence of otitis media in healthy children under five years living in low- and middle-income countries. In one review, there was only a small trial suggesting that zinc supplements might decrease the occurrence of otitis media in infants undergoing treatment for severe malnutrition, but it must be viewed with caution. Although initial results do not seem promising, it is still unclear whether zinc supplements can prevent otitis media in low-income community settings and, if so, which types of communities and which age groups may benefit [184].

#### 1.2.4. Kidney Diseases

Zinc increases the response to treatment in many infections, including urinary tract infections (UTIs). UTIs are the most common disease of the urinary system and the second most prevalent infection in children after viral influenza. Symptoms in children may be nonspecific, with weight loss, growth retardation, jaundice, and fever of unknown origin [185,186,187]. The effectiveness of zinc supplementation in children was studied by Yousefichaijan et al. [188], who highlighted a significant difference between those who received zinc supplementation and the control group regarding the duration of dysuria, urinary frequency and urgency, and recovery time (Table 2). In contrast, no significant differences were highlighted between the two groups regarding the time to resolution of fever, the time to negative urine cultures, urinary incontinence, and dribbling. Children who received zinc supplementation reported greater persistence of abdominal pain, likely due to adverse effects associated with zinc intake. As a result, the study concluded that zinc supplementation may serve as an adjunctive medication to relieve symptoms such as dysuria and frequent urination in children with urinary tract infections. Still, its use is not recommended for those experiencing abdominal pain. Similar results have been observed in studies of adults, suggesting that zinc supplementation may offer symptomatic relief and speed recovery from urinary tract infections [189]. Another frequent kidney disease in pediatric patients is nephrotic syndrome (NS), characterized by significant proteinuria, hypoalbuminemia, and generalized oedema [190]. From a histological point of view, minimal change nephropathy (MCN) emerges as the predominant histopathological subtype of infantile idiopathic nephrotic syndrome [191]. Nephrotic syndrome can recur, and the main triggers are acute respiratory infections, UTIs, diarrhea, peritonitis, and skin infections [192,193,194,195,196]. In pediatric patients, low zinc levels have been found during remission or relapse of nephrotic syndrome, regardless of serum albumin levels, probably due to urinary losses [197]. Given its well-established role in preventing acute respiratory infections, studies have suggested that zinc supplementation may reduce relapses in children with NS and promote prolonged remission [198] (Table 2). Still, the quality of the available evidence was deemed very low [199]. Children with chronic kidney disease (CKD) have alterations in tubular reabsorption that are partly responsible for low levels of circulating zinc and increased 24 h urinary zinc excretion [200]. Renal function has a linear relationship with serum zinc concentration, and zinc concentrations can accurately predict the preservation of renal function regardless of albumin levels, eGFR, age, gender, and other associated health conditions [201]. In patients with CKD, zinc deficiency may represent a risk factor for progression to ESRD [202] and for CVD due to a greater probability of abdominal aortic calcification [203,204]. The risk of CVD is also linked to the development of a fibrotic process involving not only the kidneys but also the heart, resulting in severe myocardial dysfunction [205,206] (Table 2). Zinc supplementation results in a modest but noteworthy improvement in nutritional status among children and adolescents diagnosed with chronic renal failure [207]. The KDOQI recommends monitoring dietary zinc intake according to the DRI for children and adults and recommends assessing serum zinc levels before supplementation [202]. Supplementation shows promise in mitigating nutritional deficits and slowing down fibrotic processes, but further research is needed to clarify its impact on renal outcomes [208].

#### 1.2.5. Endocrine Disorders

Zinc plays a crucial role in children’s growth, and ZnD can stunt growth. The role of calcium and vitamin D in bone homeostasis is well known, while that of zinc still needs to be completely clarified. ZnD may be a contributing factor to impaired growth in children with growth disorders, including those with growth hormone deficiency (GHD). ZnD should be considered when initiating human growth hormone treatment recombinant (rh-GH) [209]. However, the role of zinc supplementation in children with GHD, especially in high-income countries, remains controversial in the literature. Zinc supplementation improved growth velocity [209,210] and enhanced the action of vitamin D in bone formation and the effects of GH on bone [210,211]. It also has a stimulatory effect on osteoblastic bone formation through collagen synthesis and an inhibitory effect on osteoclastic bone resorption. Zinc enhances the anabolic effects IGF-1 in osteoblastic cells by exerting a complex network [212]. For these reasons, ZnD could decrease growth in children with growth disorders. Zinc supplementation may benefit growth parameters in children with GHD, particularly when ZnD is present, optimizing the body’s response to rh-GH therapy (Table 2). Further studies are needed to evaluate the importance of zinc supplementation in children with GHD in regions where ZnD is less prevalent or when adequate nutrition is maintained. Zinc plays a crucial role in regulating the synthesis and functioning of thyroid hormones [213]. It is involved in the synthesis of thyrotropin-releasing hormone (TRH) and its action on the pituitary gland, aiding in the production of thyroid-stimulating hormone (TSH). Additionally, zinc moderates the function of deiodinases, which control the synthesis and concentration of triiodothyronine (T3) and thyroxine (T4). Zinc ions are also present in T3 nuclear receptors [214] (Table 2). Consequently, there has been significant interest in exploring the effects of zinc supplementation on thyroid function, particularly in individuals with underlying risk factors. Key studies on the role of zinc supplementation in thyroid disorders have focused on patients with Down syndrome [215,216], who typically exhibit subclinical hypothyroidism, and children in developing countries [217]. Research has shown that zinc supplementation can normalize elevated TSH levels in these populations. Moreover, studies comparing zinc levels in patients with and without endemic goitre have revealed higher rates of zinc deficiency among those with goitre. Following zinc supplementation for six months, improvements in serum zinc levels corresponded to decreased serum TSH concentrations [217]. The relationship between zinc and thyroid hormones is bidirectional, as thyroid hormones are essential for zinc absorption, and zinc is necessary for thyroid function [218]. While zinc supplementation appears beneficial in specific groups, such as those mentioned, further well-designed studies are needed to assess its role in the general population based on these findings.

Moreover, there is some evidence that zinc supplements can be used to maintain the balance of blood fats and blood sugar levels for overweight and obese children [219]. In addition, zinc also improves endothelial function in obese pediatric patients [220], and it is known that SARS-CoV2 infection has a negative impact on obesity [221,222].

#### 1.2.6. Hematological Diseases

Multiple factors often influence the development of anemia, and although iron is the trace element most correlated with this condition, an alteration in zinc status can also cause its development. Both a deficiency and an excess of zinc, the latter being rarer, can cause anemia. Still, in the same way, a state of anemia is also responsible for an alteration in blood levels of zinc [223]. ZnD often accompanies iron deficiencies, and these trace elements are generally introduced into the diet [224]. Zinc plays an important role in erythropoiesis by acting as a catalyst in heme metabolism. It is a part of the ZFP GFi-1B structure that regulates cell proliferation and differentiation in erythropoiesis [225,226] (Table 2). Zinc deficiency at pediatric ages can also, to a lesser extent, collaborate in the induction of anemia with other nutritional deficiencies, such as those in folate, vitamin B12, vitamin B6, riboflavin, and vitamin A, or with infections or inflammation [227]. Although excessive zinc intake is rare, it can impair the absorption of copper, resulting in anemia [228]. The relationship between zinc and anemia is bidirectional. Studies on animal models suggest that zinc is redistributed from plasma and bones to the bone marrow to promote the production of new red blood cells during anemia. Therefore, it is necessary to evaluate zinc status in patients with anemia or diseases predisposing them to anemia, such as chronic kidney disease (CKD), and provide zinc supplementation if required [223]. Some studies have investigated the possible use of zinc supplementation in patients with sickle cell anemia (SCA). Data from small clinical trials in the United States and India suggest that zinc supplementation reduces infections in adolescents and adults with SCA, while a randomized clinical trial in children younger than 5 years with SCA in Uganda did not show a reduction in the incidence of serious infections or invasive infections. However, it should be considered that in the latter study, a large percentage of children remained zinc deficient despite one year of zinc supplementation. It is, therefore, clear that further studies on the effectiveness of zinc supplementation in preventing infections and serious outcomes in pediatric patients are warranted [223,229]. In conclusion, the involvement of zinc in anemia is complex, and the interaction of zinc with other factors or diseases in the context of anemia deserves great attention. In managing anemia, careful monitoring of zinc status is essential, and zinc supplementation may offer both preventative and therapeutic benefits [223].

#### 1.2.7. Neuropsychiatric Disorders 

Zinc is a crucial micronutrient for brain development. Insufficient zinc levels represent a significant environmental stressor that could potentially contribute to the pathogenesis of neuropsychiatric disorders such as autism spectrum disorder (ASD) [230], febrile seizures in children [231], and depression [232]. ASD is a group of neurodevelopmental disorders characterized by deficits in social communication and language, as well as repetitive and stereotyped behaviors. The worldwide prevalence of ASD is around 1%, which is probably underestimated [233]. Although no direct causal factor for the development of this condition has been identified, brain growth and development appear to be altered at some stage of the child’s development [234]. Numerous genetic and environmental causes appear to contribute to the development of ASD. The main genes involved are implicated in synaptic development, function, and activity-dependent plasticity [235]. Other potential etiological pathways associated with ASD include immune system dysfunction and brain–gut dysbiosis. Zinc deficiency may be an etiological factor linking all these pathways [230]. Zinc supplementation increases IGF-1 levels [236,237], which can reduce neuronal excitability and improve oligodendrocyte function in preventing myelination defects [238]. Furthermore, IGF-1 can enhance oxytocin secretion, promote cell survival and growth, and regulate several cellular processes such as apoptosis [238]. Oxytocin (OXT) and vasopressin (AVP), two neuropeptides recognized primarily for their role in social behavior, are also the subject of an intense investigation regarding their involvement in the development of ASD. However, the exact mechanisms remain incompletely understood. Oxytocin receptors (OXTRs) and vasopressin receptors (V1aR and V1bR) are found in large quantities in brain regions implicated in social cognition and emotional regulation [239]. These receptors mediate social bonding, empathy, trust, and stress responses. In children with ASD, the alteration of these receptors’ expression, distribution, or functioning has been attributed to the development of alterations in social behavior and emotional processing [239,240]. Studies have reported changes in genetic sequences and epigenetic modifications of OXTRs and vasopressin receptor genes in individuals with ASD. Zinc appears to modulate the social and behavioral effect of neuropeptides by interfering with the function of transcription factors and modulating the effect of vasopressin on the hypothalamic–pituitary axis. Furthermore, it limits the lifespan of OXT and AVP by contributing to the function of leucyl-cystinyl aminopeptidase (LNPEP), which inactivates vasopressin and oxytocin [230] (Table 2). However, all these data potentially linking zinc to ASD through an effect on neuropeptides come from preclinical studies with different animal models for ASD, while clinical studies appear rather controversial [241,242]. Certainly, the impact of zinc deficiency on the brain of the fetus, newborn, and child needs to be further studied to understand better at what stage, in utero due to maternal deficiency or postnatally due to environmental factors, ZnD has a major impact on the brain of the fetus, newborn, and child. Several studies have explored the relationship between zinc levels and febrile seizures. Children with febrile seizures had significantly lower serum zinc levels compared to healthy controls [243]. This suggests that zinc deficiency may predispose children to febrile convulsions, possibly due to its role in modulating neuronal excitability (Table 2). Furthermore, zinc supplementation has shown promise in reducing the frequency and severity of febrile seizures. Fallah et al. demonstrated that children receiving zinc supplementation experienced fewer febrile episodes than those receiving a placebo [244]. This suggests that optimizing zinc status could lower the risk of febrile seizures in susceptible children. The mechanisms underlying the protective effect of zinc against febrile seizures are not fully understood but may involve its anti-inflammatory and antioxidant properties. Fever-induced inflammation and oxidative stress contribute to neuronal hyperexcitability, potentially triggering seizures. By modulating these processes, zinc may help stabilize neuronal activity and reduce the likelihood of seizure occurrence during febrile episodes. Emerging research has shed light on the potential connection between zinc deficiency and depression, highlighting the importance of adequate zinc levels for mental well-being. In the brain, zinc concentrations are highest in the hippocampus and amygdala [245,246]. Zinc is a key cofactor in the synthesis and metabolism of neurotransmitters; it also contributes to the functioning of brain and neural structures by modulating synaptic transmission and acting as an endogenous neuromodulator of important receptors such as alpha-amino-3-hydroxy-5-methyl-4-isoxazole-propionic acid (AMPA), gamma-aminobutyric acid (GABA), and N-methyl-D-aspartate (NMDA) [245]. Various mechanisms implement the antidepressant effect of zinc. One potential mechanism is its inhibitory action against NMDA receptors either by direct receptor antagonism [247,248] or by the indirect inhibition of NMDA receptors through the activation of AMPA receptors [249]. In contrast, zinc deficiency increases glutamatergic neurotransmission through NMDA receptor activation [245]. Evidence suggests that just such an increase in glutamatergic neurotransmission may be associated with depression and neurotoxicity [250], so NMDA receptors have been identified as a possible therapeutic target in studies on the treatment of depression [251,252]. In addition, as zinc is a modulator of excitatory (glutamatergic) and inhibitory (GABAergic) amino acid neurotransmission [249], alterations in its homeostasis at the cellular level may impair the nervous system’s ability to adapt to structural and functional changes in response to environmental changes and novel experiences, which may contribute, in the long term, to the development of psychiatric diseases [253,254]. There is evidence that zinc deficiency may increase the levels of proinflammatory cytokines, including IL-6 and TNF-α [255,256]. The elevation of proinflammatory cytokines may favor brain damage and changes in serotonin functions in the brain, predisposing an individual to depression [257]. However, the relationship between zinc levels and proinflammatory cytokines in the aetiology of depression appears to be bidirectional since the increase in cytokines seen in patients with depression may also contribute to a reduction in zinc levels [258]. Zinc is also involved in the regulation of the expression of brain-derived neurotrophic factor (BDNF), which plays an important role in neuroplastic processes, neuronal tropism, memory, learning, and memories [259,260], and its defect can contribute to the development of depressive disorders [261] (Table 2). In depressed patients, serum zinc levels are lower than in healthy controls; furthermore, an inverse relationship has been highlighted between serum zinc levels and the severity of depression [253], but also between the levels of zinc consumed and the risk of developing it [262]. Some studies appear to demonstrate that zinc supplementation can lead to a reduction in depressive symptoms in individuals with a clinical diagnosis of major depression treated with antidepressant therapy [232]. Because of the higher prevalence of depression in adults than in children and because of the lesser chance of conducting studies on pediatric patients, there is more evidence in the literature on the role of zinc supplementation in depression in adults. To date, few but promising studies on children and adolescents show similar results to studies on adults. In a cross-sectional study conducted by Amani et al. significantly lower levels of serum zinc were observed in female students with depression in comparison with non-depressed matched subjects. Moreover, their study showed an inverse correlation between dietary zinc intake and serum zinc levels with depression scores [263]. In addition, a study on a sample of children at risk for zinc deficiency in Guatemala showed an association between the increase in serum zinc concentrations and the decrease in internalizing symptoms such as depression and anxiety [264]. However, research evaluating the effect of zinc supplementation on depression is still poor, especially in children and adolescents, and further studies are needed to confirm the beneficial effect of zinc in these patients.

**Table 2:** Role of zinc in organs and systems.

Organs and Systems	Role of Zinc
Respiratory system	
SARS-CoV2	Enhanced immune response, reduced risk of co-infections [145], increased susceptibility of bacteria to killing by neutrophils [147], inhibition of biofilm formation [149], increased ciliary length and beating [153,154], and regulation of tight junction (ZO-1 e Claudin1) [155]. Inhibited RNA polymerase activity [156], modulated ACE2 receptors [4,157,158], production of IFN-α by lymphocytes and antiviral enzymes [159].
Community-acquired pneumonia	Antibacterial and anti-inflammatory properties, regulated tissue growth, and reduced stimulus to the production of toxins by microorganisms [95,97].
Cystic fibrosis	Promoted growth, sexual maturation [109,110], immune response (enhanced IL-2 production, NK cells, and thymulin activity) [102,106], and appetite. Controversial improvement of lung function [108,111,117].
Chronic lung disease	Improved growth [136].
Gastrointestinal system	
Growth	Increased ghrelin and IGF-1 secretion [167], hunger, and food intake [169,170]. Also, increased growth regardless of food intake (protein synthesis) [167,171,174].
Gastrointestinal infections	Increased mucosa regeneration, protein synthesis, and apoptosis inhibition [177,179]. Enhanced increased villus height, ratio of villus height to crypt depth, and decreased intestinal permeability [177,179]. Improved flora diversity and reduced proinflammatory cytokines and mediators (IFN-α, NO, IL-2, IL-6, and TNF) [180,181].
Kidney	
Urinary tract infections	Reduced duration of dysuria, urinary frequency, urgency, and recovery time [188].
Nephrotic syndrome	Reduced relapse [198,202].
Chronic kidney disease	Reduced progression to ESRD [202]. Defense against phosphate-triggered calcification of the abdominal aorta [203,204]. Reduced fibrotic processes [208].
Endocrine system	
Growth hormone deficiency	Improved growth velocity [209,210], action of vitamin D in bone formation, and effects of GH on bone [210,211], optimizing the body’s response to rh-GH therapy [209]. Stimulated collagen synthesis and inhibited effect on osteoclastic bone resorption. Enhanced effects of IGF-1 in osteoblastic cells [212].
Dysthyroidism	Involved in TRH, TSH, T3, and T4 synthesis [213,214].
Hematopoietic system	
Anemia	Constituent of ZFP GFi-1B that regulates cell proliferation and differentiation in erythropoiesis [225,226].
Brain	
Autism spectrum disorder	Increased IGF-1 levels reducing neuronal excitability and improving oligodendrocyte function in preventing myelination defects [236,237]. IGF-1 can also enhance oxytocin secretion, promote cell survival and growth, and regulate several cellular processes such as apoptosis [238,239]. Modulated social and behavioral effect of OXT and AVP [239]. Reduced OXT and AVP lifespan contributing to the function of LNPEP [230].
Febrile seizure	Modulated neuronal excitability [244].
Depression	Contributed to synthesis and metabolism of neurotransmitters. Modulated excitatory (glutamatergic) and inhibitory (GABAergic) amino acid neurotransmission [245,249]. Reduced proinflammatory cytokine relapse and changes in serotonin functions [257]. Enhanced BDNF production, which plays an important role in neuroplastic processes, neuronal tropism, memory, learning, and memories [259,260].

ZO-1: zonula occludens-1; ACE: Angiotensin Converting Enzyme; IFN: interferon; IL: interleukin; NK: natural killer; IGF-1: insulin-like growth factor-1; NO: nitric oxide; TNF: tumor necrosis factor; ESRD: End-Stage Renal Disease; GH: growth hormone; rhGH: recombinant human growth hormone; TRH: thyrotropin-releasing hormone; TSH: thyroid-stimulating hormone; T3: thyroxine; T4: tetraiodothyronine; GFi-1B: growth factor independence 1B; OXT: oxytocin; AVP: vasopressin; LNPEP: leucyl-cystinyl aminopeptidase; GABA: gamma-aminobutyric acid; BDNF: brain-derived neurotrophic factor.

## 2. Future Directions

Although the molecular mechanisms through which zinc can influence the inflammatory and immune states and the oxidative balance in the human body are now known, further studies are necessary to evaluate its effectiveness as a combined therapy in specific clinical contexts. It is important to conduct large-scale clinical trials focused on zinc supplementation in different populations with different risk factors or underlying clinical conditions to evaluate its real effectiveness in therapeutic management. These studies should evaluate the effectiveness and safety of zinc supplementation as an adjunctive therapy in combination with standard treatments, evaluating its impact on disease severity, duration, and relapse rates. Such studies should aim to delineate the most effective methods for identifying zinc deficiency states and establish the optimal dosage and duration of zinc supplementation. These determinations will be based on age, disease severity, and nutritional status. The goal is to maximize therapeutic benefits while minimizing potential adverse effects. Subsequently, the prolonged impact of zinc supplementation beyond the acute phase of the disease should also be evaluated to understand its role in managing and preventing the disease. Finally, in developing countries where zinc deficiency is critical in the pediatric population, educational campaigns and nutritional interventions should be implemented to reduce the impact of this deficiency on children’s health and the nation’s development.

## 3. Conclusions

This comprehensive review thoroughly dissects the multifaceted role of zinc in children’s physiology and underscores its importance in maintaining overall health in developed countries. Due to its anti-inflammatory, antioxidant, and immune function, zinc emerges as a crucial micronutrient with far-reaching implications for well-being. The ability of zinc to modulate NF-κB activity helps to balance immune reactions and prevent excessive inflammation. Moreover, the downstream effects of zinc on immune cells like basophils, leukocytes, and mast cells contribute to its anti-inflammatory properties, potentially offering therapeutic benefits in managing allergic reactions and airway hyper-responsiveness. Additionally, its antioxidant activity underscores its role in neutralizing harmful free radicals, thereby protecting cells from oxidative damage and reducing the risk of chronic diseases. Moreover, zinc enhances the ability to mount effective immune responses against pathogens by contributing to lymphocyte maturation, cytokine production, and apoptosis regulation. In the context of SARS-CoV-2 infection, zinc supplementation demonstrates a potential role in enhancing mucociliary clearance, inhibiting viral replication, and modulating inflammatory responses. Moreover, the antibacterial properties of zinc, observed in CAP, further underscore its potential in managing respiratory infections in pediatric patients. Zinc supplementation can reduce the severity and duration of pneumonia, potentially by enhancing immune function and reducing inflammation. Additionally, the role of zinc in maintaining respiratory epithelial barrier function and regulating tight junction proteins highlights its importance in preventing infections. Furthermore, zinc supplementation has been shown to shorten the duration of gastrointestinal infections and prevent their progression in the pediatric population. The ability of zinc to aid intestinal mucosal regeneration and improve barrier function contributes to its therapeutic effects in such infections. Zinc supplementation has demonstrated symptomatic relief in kidney diseases like UTIs, the most common disease of the urinary system and the second most prevalent infection in children after viral influenza, by alleviating symptoms such as dysuria and urinary frequency, albeit with considerations for potential adverse effects like abdominal pain. Furthermore, the role of zinc in preventing acute diseases, particularly those of the respiratory system, has been shown to reduce the incidence of relapses in patients with nephrotic syndrome. In addition to its role in the prevention and treatment of acute infections, zinc is also important as a support in the prevention and treatment of numerous chronic conditions in pediatric patients. Zinc is a crucial micronutrient for developing the body and the brain. Low levels of zinc have been found in patients suffering from cystic fibrosis, CLD, GHD, and alterations in thyroid function, where zinc plays an important role in counteracting the stunted growth typical in these pathologies. Zinc is important in promoting food intake and weight gain by stimulating the secretion of ghrelin and IGF-1. However, the role of zinc in growth involves some mechanisms independent of food intake that still need to be completely cleared. Numerous studies have also highlighted how low zinc levels are related to greater severity of CKD and depression and an increased risk of febrile seizure. Therefore, maintaining an adequate zinc intake to ensure optimal levels is important for an individual’s overall health. When addressing nutritional deficits arising from malnutrition, restrictive diets, inadequate absorption, or excessive losses, zinc supplementation could emerge as a possible adjunctive therapy in managing various pathologies, whether acute or chronic.

## Data Availability

Not applicable.
